# Exceptional endemicity of Aotearoa New Zealand biota shows how taxa dispersal traits, but not phylogeny, correlate with global species richness

**DOI:** 10.1080/03036758.2023.2198722

**Published:** 2023-04-20

**Authors:** Mark John Costello

**Affiliations:** Faculty of Biosciences and Aquaculture, Nord University, Bodo, Norway

**Keywords:** Biogeography, biodiversity, marine, freshwater, terrestrial, body size

## Abstract

Species’ with more limited dispersal and consequently less gene flow are more likely to form new spatially segregated species and thus contribute disproportionally to endemic biota and global species richness. Aotearoa New Zealand has exceptional endemicity, with 52% of its 54,000 named species endemic, including 32%, 39% and 68% for freshwater, marine and terrestrial environments respectively. The lower endemicity of freshwater biota (excluding insects) is attributed to their need to disperse between habitats that are temporary on evolutionary timescales. The percent endemicity of higher taxa (Order to Kingdom), a measure of phylogenetic relationships, was not correlated with regional and global species richness. However, there was a positive correlation between endemicity and species richness across dispersal trait groups based on their environment, typical body size, mobility (including flight), and if marine, whether pelagic or benthic. Typically flighted taxa had high endemicity contrary to the dispersal-endemicity hypothesis, but reflecting exceptional isolation by distance and time, and reduced flight ability as occurs on islands. It is proposed that the high richness and endemicity of mobile macrofauna is caused by a combination of niche specialisation opportunities and predation limiting dispersal respectively. Thus, dispersal traits better predicted endemicity and global species richness than phylogeny.

## Introduction

Kingdoms, phyla, orders and other higher taxa vary greatly in species richness as a consequence of their evolutionary history, likelihood of new species forming, and relative extinctions. Taxa that have high dispersal and gene flow will be less likely to form new species than taxa where populations become separated by geographic, environmental, ecological or biological barriers. Thus, widely dispersing taxa will have relatively low rates of endemicity. In contrast, taxa with high rates of species endemicity, that is species restricted in their geographic distribution, should have a higher number of species globally.

**Figure 1.**
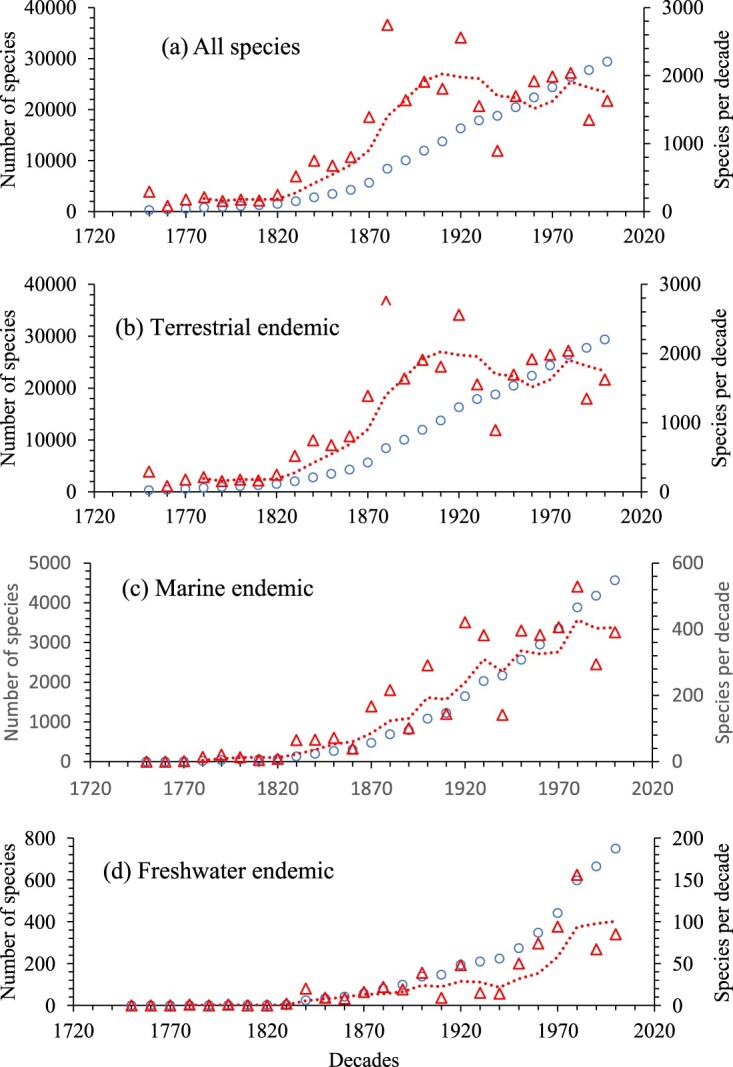
The number of species described from 1757 to 2010, in total (blue circles) and per decade (red triangles with four-point moving average trend line) for (**A**) all species, and all endemic (**B**) terrestrial, (**C**) marine and (**D**) freshwater species in New Zealand. Note that 45% of all listed species are not plotted because the database lacked their year of description; and that scales vary.

Aotearoa New Zealand is exceptional in being a long isolated continental land mass, having a large proportion of endemic species, and being a relatively well-sampled country (Hughes et al. 2021). It also has an inventory of its freshwater, marine and terrestrial fauna and flora, with species origins noted as endemic or exotic (i.e. deliberately and accidentally introduced by human activity) (Gordon 2009, 2010, 2012), and reviewed by Gordon (2013). The peak period of terrestrial and thus overall species descriptions was around the beginning of the nineteenth century but much later for marine species (Figure 1A–C), similar to global trends (Costello et al. 2012). That the rate of endemic species description has remained high, at least until the 1980s for species in all three environments (Figure 1B–D), emphasises the high endemicity of the biota. If the number of described species is added to estimates of the number of new species in specimen collections, then 85% (across phyla average 77%, median 81%) of species have been described (Gordon 2013). Yet if the estimates of uncollected species in Gordon (2009, 2010, 2012, 2013) in all Kingdoms are accounted for then only 40% (across phyla average 45%, median 44%) of New Zealand species had then been described. Considering these estimates were speculative, made over a decade ago, do not account for as yet to be recognised synonyms which may be over 20% of species names, and that globally two thirds of all estimated species have been described across taxa in marine, terrestrial and freshwater environments, including parasites (reviewed Costello 2020), it is likely that at least half of all species occurring in the New Zealand have been named. With respect to the present paper, the most important aspect of these trends is that the high endemicity of New Zealand’s native species does not appear to be an artefact of insufficient sampling. Indeed, the proportion of New Zealand’s species that are endemic appears to be the highest for any country in the world. The only region known to have similarly high marine endemicity is Antarctica (Costello et al. 2010; Gordon et al. 2010). Therefore, this inventory provides an opportunity to see how relative endemicity correlates with species richness at regional and global scales.

Costello and Chaudhary (2017) reviewed factors likely to lead to greater isolation and thus endemicity and species richness. Isolation is generally greater for terrestrial and freshwater than marine species due to the continuous ocean environment. Endemicity is thus higher in terrestrial than marine environments, and so is the number of species, The Bass-Becking hypothesis asserts that microscopic organisms will be more cosmopolitan than larger organisms because of their great abundance, low probability of extinction, spores that can survive years and centuries, and ease of passive dispersal in air, water and on animals, by comparison with larger organisms. Individual large mobile animals (megafauna), such as birds, mammals, reptiles and fish, can disperse across continents and oceans. Thus, in the ocean the most cosmopolitan taxa are the pelagic taxa with the largest and smallest body sizes (Costello et al. 2017). Because mobility provides species with increased ability to find scattered habitats, food sources and reproductive partners, and to avoid predation, it may provide opportunities for speciation into more diverse ecological niches than available to sessile and sedentary species (reviewed in Costello and Chaudhary 2017). Thus, it is hypothesised that (1) mobility may reduce endemicity but increase species richness. Furthermore, it is hypothesised that percent endemicity and species richness will be less in:

 2. marine than terrestrial and freshwater environments;
 3. microscopic than larger organisms;
 4. megafauna than macrofauna;
 5. flighted than species without active aerial dispersal;
 6. marine pelagic (open water living) than benthic (seabed living) species;

A lack of support for these hypotheses could reflect the quality of the underlying data available on species endemicity and richness, and/or that these characteristics are not good indicators of species gene flow. For example, classifying higher taxa into body size classes based on their adult life-stage does not account for the longevity and dispersal of microscopic life-stages, whether spores, eggs, or larvae (Costello et al. 2015a, 2015b). Colonial organisms like many species of cnidarians, sponges and bryozoans live some time as microscopic organisms but form large colonies. Thus, the present analysis explores whether general patterns exist that could be improved upon when more detailed classifications of species traits are available.

## Methods

### Source data

The dataset was obtained in two parts, from the online New Zealand Organisms Register (NZOR) (Anonymous 2018) and an original digital version for animals only whose original source was the three volumes edited by Gordon (2009, 2010, 2012). It includes not only described species, but candidate species yet to be described and which occur in specimen collections. These undescribed taxa are assumed to be likely to be described as new species and thus counted here as equal to named species so the dataset is a more accurate indicator of the total biotic diversity. These sources were compared with each other and the summary published by Gordon (2013). Differences between datasets arose due to alternative taxonomic classifications, including different hierarchical arrangements of phyla and classes; e.g. Charophyta within Chlorophyta in NZOR. Following Gordon (2013), Anthocerotophyta and Marchantiophyta are included with Bryophyta, and Dinophyta (Dinoflagellata) with Myzozoa (Miozoa), and Radiozoa with Foraminifera. Minor errors in attribution of species as living and fossil (e.g. most Hemichordata in NZOR were fossil species), and by marine, terrestrial and freshwater environment (e.g. Pycnogonida mistakenly included as terrestrial), were corrected. Instances of a species being recorded twice with different spellings of authority names occurred, and instances of higher taxa being included in the species names field were removed prior to analysis. Curiously, the only Phylum that was in the source volumes but not available in the digital data was that comprising the three native (not endemic) species of penis worms, Priapulida. The final data used is available as Supplementary Material.

Marine species were recorded for the New Zealand Exclusive Economic Zone and thus range from sub-tropical latitudes to sub-Antarctic islands, and the intertidal to deep-sea. Only taxa classified by endemicity and environment were included in this analysis. While the numbers of exotic species are shown per taxon, they are not included in the analyses.

The number of species and percent endemicity were provided at different levels in the taxonomic hierarchy as the available data permitted (Table 1). Phyla with less than five species were not tabulated, namely: Dicyemida (6 marine endemic species), Echiura (7 spp), Gastrotricha (1 freshwater, 4 marine), Nematomorpha (3 freshwater and 1 marine endemic, 1 native freshwater), Phoronida (3), Loricifera (4 marine), Orthonectida (1 marine), Archaea (5), Glaucophyta (1 freshwater). Neither were the Charophyta due to uncertainty in their classification (29 spp in NZOR included in Chlorophyta but 527 reported in Gordon (2013) with 43 endemic). The total fungi present is similar in NZOR and Gordon (2013), but richness of Oomycota (Pseudofungi) varied (307 vs 161) and the proportions in each environment were not captured sufficiently in NZOR for environmental comparisons.

**Table 1.** The number of marine, freshwater and terrestrial species classified as endemic (end), exotic (exot) and indigenous (ind) in the New Zealand Organism Register (NZOR). The total is sometimes greater than the sum of species per environment because additional species are not unambiguously associated with one environment or were not assigned to an environment in the database.

Taxon*	Freshwater			Marine			Terrestrial			Total
	End	Exot	Ind	End	Exot	Ind	End	Exot	Ind.	
**Animalia**	**751**	**104**	**686**	**5,924**	**126**	**6,547**	**13,231**	**2,090**	**1,646**	**33,705**
Annelida	**24**	27	7	**242**	19	515	**163**	29	8	1,054
Arthropoda	**575**	42	101	**926**	33	1,815	**12,035**	1718	1198	20,142
Brachiopoda				**17**		21				38
Bryozoa			8	**570**	24	342				948
Chaetognatha						14				14
Chordata	**21**	22		**201**	4	969	**79**	132	33	2,101
Cnidaria		2	12	**252**	22	739				1,029
Ctenophora				**5**		14				19
Echinodermata				**236**		380				622
Gnathifera			448			41			1	496
Hemichordata				**1**		250				269
Kamptozoa				**2**	1	5				8
Kinorhyncha				**6**		13				19
Mollusca	**77**	8	1	**2,906**	10	639	**805**	31	5	4,600
Nematoda				**9**		121	**45**	150	380	732
Nemertea	**2**		2	**19**		10	**4**		1	39
Onychophora							**11**		1	12
Platyhelminthes	**23**	3	50	**89**	2	208	**89**	30	19	521
Porifera	**1**		4	**329**		375				709
Sipuncula				**2**		19				26
Tardigrada	**25**		51			4				88
Tunicata				**121**	11	60				192
**Bacteria**						**39**	**13**	**1**	**608**	**674**
**Chromista**	**44**	**3**	**857**	**60**	**13**	**1,020**	**10**	**129**	**154**	**4383**
Bigyra		2	1			8		1		13
Cercozoa	**4**		18	**3**		7			33	83
Ciliophora	**38**		80	**8**	1	62		32	103	334
Cryptophyta			7	**1**		9				19
Foraminifera				**120**	3	1002				1131
Haptophyta			2	**2**		95				99
Heliozoa	**1**		11			1			1	15
Myzozoa			40	**2**		202	**10**	96		383
Ochrophyta	**1**	1	697	**46**	12	731			17	1,505
Fungi**	**2**		**9**	**1**		**1**		**5,561**	**1**	**7,492**
**Plantae**			**21**	**30**	**8**	**107**	**2542**	**1901**	**2415**	**7,042**
Bryophyta							**406**	46	779	1,231
Chlorophyta	**50**	6	882	**29**	8	106			31	1,112
Rhodophyta			20	**179**	13	268				470
Tracheophyta				**1**		1	**1907**	1807	488	4,203
**Protozoa**	**3**	**3**	**139**	**11**		**64**	**9**	**18**	**274**	**521**
Grand Total	800	110	1,712	6,026	147	7778	15,805	9,700	5,098	53,817

The number of accepted species per taxon was obtained from the World Register of Marine Species (WoRMS) for marine and some associated taxa (Costello et al. 2013a, Horton et al. 2022). Several taxa in WoRMS include all their freshwater and marine relatives, including the crustaceans and molluscs. These data were cross-checked against recorded and estimated numbers of species in taxa in the Catalogue of Life (Bánki et al. 2022), including for freshwater and terrestrial species. Where differences in the taxonomic classifications between these three sources made comparisons questionable these data were excluded from the analyses. With two million species listed, the CoL is an almost complete checklist of all accepted species (Costello et al. 2022). Thus, the recorded number of species in most taxa approximates the number of accepted species, notwithstanding that new species are still being named, names are being synonymised and classifications revised (Costello et al. 2013a, Costello 2020).

### Biological traits

In the dataset, all birds and insects were classified as terrestrial, even though some birds are closely associated with freshwater and/or marine environments, and several orders of insects live most of their lives in freshwaters. Because only 2% of the Plantae species were classified by environment, comparisons across environments were dominated by animals, microbes (i.e. Bacteria, Archaea, Protozoa) and Chromista. Taxa were attributed a typical body size as microscopic if 1 mm or less, megafauna if typically > 10 cm and macroscopic in-between these values, following Costello et al. (2013b). As many marine taxa have both pelagic and benthic life-stages, and data on their relative duration and significance are not available at species level, those that were almost entirely pelagic and benthic were compared: pelagic being Chaetognatha, Ctenophora, Cubozoa, Mammalia, Reptilia, Scyphozoa; and benthic the peracarid crustaceans which lack planktonic larvae, namely Amphipoda (excluding 101 species in New Zealand associated with pelagic hosts), Cumacea, Isopoda and Mysida (Table 2). All birds and insects were classified as ‘flighted’ because it is assumed their first colonists of New Zealand could fly, although this ability varies today. The Phthirptera were included as flighted insects because their hosts, birds, fly. However, the also flightless flea parasites, Siphonaptera, were not classified as flighted because about one third of species in New Zealand parasitise mammals (excluding bats) so the estimate of flighted species is conservative. The classification kept the Entognatha, namely the wingless arthropods, Collembola, Diplura, Protura and Thysanura separate from the Insecta. Most vertebrates, arthropods and annelids were classified as mobile, although there are exceptions in most higher taxa (Table 2).

**Table 2.** Taxa grouped into five categories of percent endemicity. Taxa consider microscopic, including colonial species with microscopic individuals, are in red. Marine taxa which are only pelagic or benthic are in blue with the benthic underlined. Most species in other underlined taxa are considered sedentary. Taxa are listed in order of endemicity. Note that all Aves and Pinnipedia Carnivora (seals and sea lions) Mammalia were classified as terrestrial, while all Cetacea were marine.

%	Freshwater	Marine	Terrestrial
>80	Neuroptera, Thysanoptera, Amphipoda, Bathynellacea, Isopoda, Cyclopoida, Chordata, Actinopterygii, Bivalvia, Mollusca, Gastropoda, Diptera, Malacostraca, Arachnida, Coleoptera, Insecta, Hemiptera, Arthropoda, Odonata, Anaspidacea, Clitellata	Myzostomida, Cephalocarida, Dermaptera, Lepidoptera, Thysanoptera, Ibliformes, Pygophora, Amphioxi, Dicyemida (Rhombozoa), Aplacophora, Monoplacophora, Nematomorpha, Orthonectida, Apusozoa, Diptera, Scaphopoda, Cumacea, Polyplacophora, Bivalvia, Gastropoda, Mollusca, Coleoptera	Chilopoda, Diplura, Archaeognatha, Blattodea, Dermaptera, Ephemeroptera, Mantodea, Neuroptera, Phasmatodea, Plecoptera, Strepsiptera, Thysanoptera, Trichoptera, Maxilopoda, Harpacticoida, Ostracoda, Pauropoda, Symphyla, Reptilia, Myzozoa, Metamonada, Coleoptera, Mollusca, Gastropoda, Diplopoda, Diptera, Orthoptera, Lepidoptera, Amphipoda, Clitellata, Insecta, Annelida, Hemiptera, Malacostraca, Isopoda, Onychophora, Amphibia, Arthropoda, Animalia, **Grand Total,** Collembola, Platyhelminthes
60	Annelida, Decapoda, Nematomorpha, Ostracoda, Maxilopoda	Isopoda, Clitellata, Tunicata, Mysida, Myxini, Nemertea, Scalpelliformes, Bryozoa, Euglenozoa	Nemertea, Isoptera, Siphonaptera, Thysanura, Arachnida, Chordata, Hymenoptera, Protura
40	Harpacticoida, Animalia, Polychaeta, Nemertea	Asteroidea, Amphipoda, Akentrogonida, Dendrogastrida, Staurozoa, Fungi, Tracheophyta, Malacostraca, Animalia, Porifera, Actiniaria, **Grand Total**, Perviata, Pycnogonida	Psocoptera, Mammalia
20	Tardigrada, Ciliophora, Platyhelminthes, **Grand Total**	Echinodermata, Sessilia, Arthropoda, Cephalopoda, Echinoidea, Spionida, Annelida, Myxozoa, Kinorhyncha, Polychaeta, Siphonostomatoida, Cercozoa, Platyhelminthes, Decapoda, Ophiuroidea, Harpacticoida, Holothuroidea, Stomatopoda, Kentrogonida, Kamptozoa, Chondrichthyes, Gorgonacea, Hydrozoa, Ctenophora, Insecta, Cnidaria, Crinoidea, Terebellida, Corallimorpharia, Plantae, Antipatharia, Chlorophyta, Alcyonacea	Bacteria, Phthiraptera, Aves
<20	Porifera, Percolozoa, Cercozoa, Fungi, Phthiraptera, Branchiopoda, Choanozoa, Heliozoa, Chromista, Protozoa, Ochrophyta, Bryozoa, Cnidaria, Hydrozoa, Myxozoa, Gnathifera, Rotifera, Cryptophyta, Dinophyta, Myzozoa**,** Plantae, Rhodophyta, Amoebozoa, Euglenozoa	Maxilopoda, Scyphozoa, Chordata, Actinopterygii, Ostracoda, Protozoa, Scleractinia, Tanaidacea, Ciliophora, Cryptophyta, Sipuncula, Nematoda, Ochrophyta, Lepadiformes, Chromista, Amoebozoa, Calanoida, Cyclopoida, Phthiraptera, Myzozoa, Hemichordata, Lobatocerebrida, Arachnida, Branchiopoda, Trichoptera, Euphausiacea, Leptostraca, Lophogastrida, Laurida, Monstriloida, Mormonilloida, Chaetognatha, Cephalasipidomorphi, Mammalia, Reptilia, Cubozoa, Ceriantharia, Pennatulacea, Zoanthidea, Echiura, Gnathifera, Acanthocephala, Gnathostomulida, Rotifera, Loricifera, Phoronida, Tardigrada, Bacteria, Bigyra, Dinophyta, Heliozoa, Choanozoa	Nematoda, Chromista, Protozoa, Amoebozoa, Polychaeta, Decapoda, Gnathifera, Acanthocephala, Cercozoa, Ciliophora, Heliozoa, Ochrophyta, Fungi, Plantae, Chlorophyta, Tracheophyta, Choanozoa, Euglenozoa, Percolozoa

## Results

### Richness

Of the total 54,000 species, most were animals (63%), invertebrates (60%) and arthropods (38%) (Table 1). Molluscs and chordates (mostly fish) comprised 9% and 4% respectively. There were similar proportions of Plantae, fungi and Chromista (14%, 13%, 8%) and few prokaryotes (Bacteria, Archaea, Protozoa, 2%) (Table 1).

**Figure 2.**
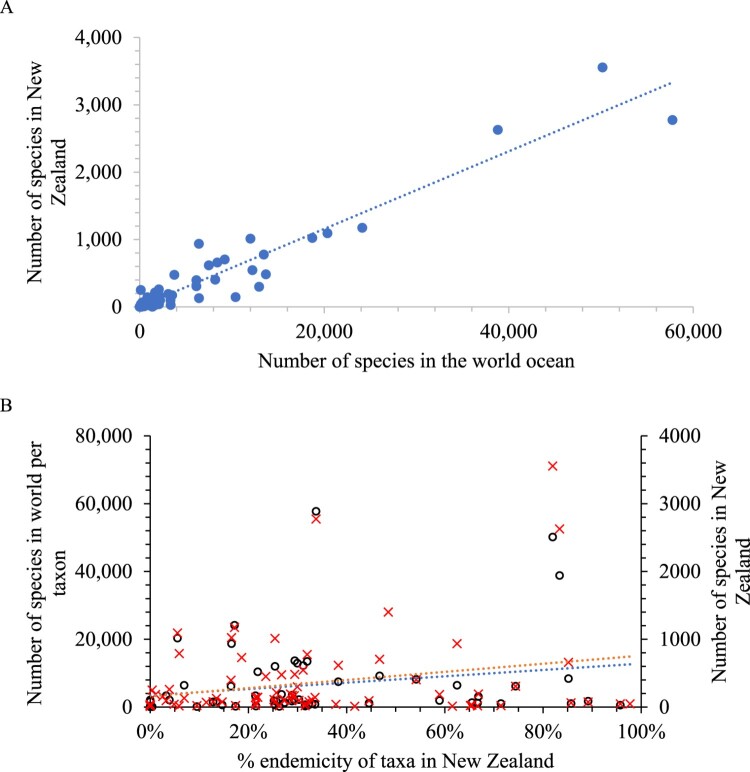
(**A**) The total number of marine species recorded in New Zealand was highly correlated (R^2^ 0.92) with the number known in the world for the higher taxa (Table 1). Even when the three most species rich taxa are excluded the correlation was still high (R^2^ 0.86). (**B**) The relationship between the number of marine species globally (red X symbols) and in New Zealand (hollow circles symbols) to percent endemicity of the higher taxa (excluding samples with less than ten species). Neither of these variables was well correlated with the percent endemicity in New Zealand (R^2^ 0.05) or globally (R^2^ 0.07).

**Figure 3.**
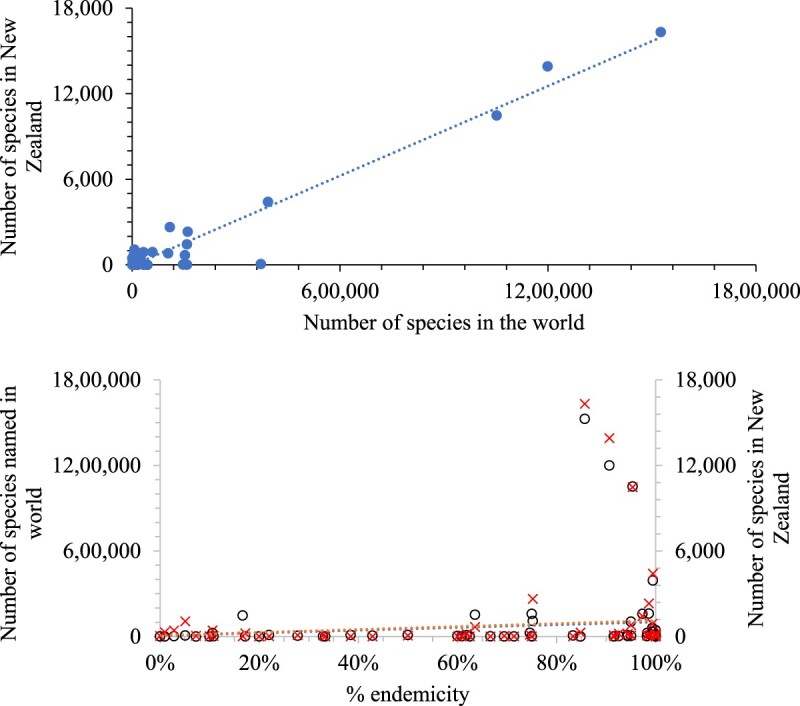
The total number of freshwater and terrestrial species recorded in New Zealand is highly correlated with the number known in the world (upper plot, R^2^ 0.95). When the three most species rich taxa are excluded the correlation is still strong (R^2^ 0.79). However, neither of these variables is well correlated with the percent endemicity in New Zealand (red X symbol) and globally (hollow circle symbol) (both R^2^ 0.01) (lower plot, excluding samples with less than ten species).

The number of species per taxon in New Zealand was highly correlated with the number globally for both marine, and for terrestrial and freshwater combined (Figures 2 and 3). However, there are differences. Excluding fungi, because 74% are introduced species but were not classified as such in the present database, the percentages for the world and New Zealand are: 78% and 65% of species are animals, 64% and 38% arthropods, 54% and 20% insects, 19% and 14% plantae and reversed with 1% and 8% Chromista. The relatively higher proportion of Chromista may be due to marine taxa comprising 30% of the New Zealand biota but 12% of the global biota.

Comparing across environments, the terrestrial harboured 65%, marine 30% and freshwater 5% of all species. However, while most and the greatest proportion of introduced species were terrestrial at 32%, fewest were marine at 1% (Table 1).

### Endemicity

There was no correlation between percent endemicity and total species richness per taxon in New Zealand, globally, nor in marine and terrestrial environments (Figures 2 and 3). Thus, percent endemicity per taxonomic (i.e. phylogenetic) group does not help predict species richness at regional or global scales.

**Figure 4.**
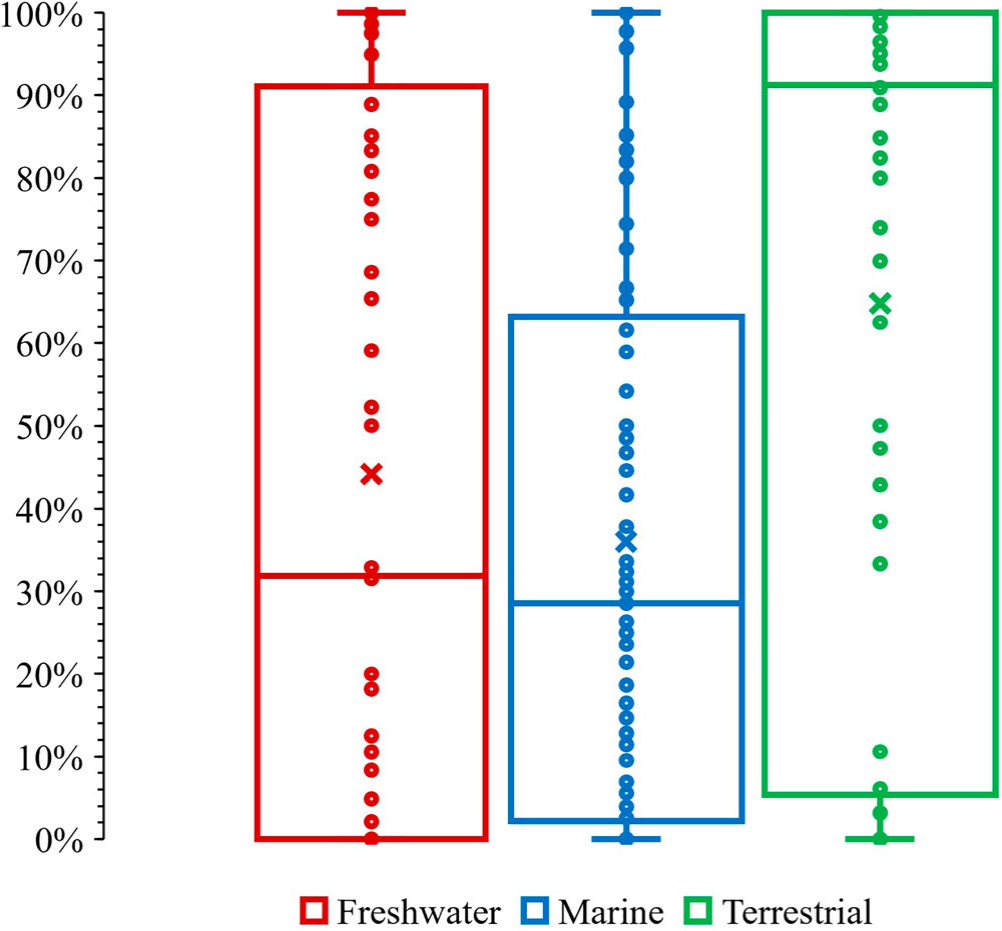
The distribution of percent endemicity across freshwater, marine and terrestrial taxa recorded in New Zealand, showing the mean (x), median (horizontal bar), range and upper and lower quartiles (box), where each data point is a taxon.

Generally, terrestrial taxa had higher endemicity and marine the lowest (see mean and quartile distributions in Figure 4). Within each environment there were taxa with very low and very high endemicity (Figure 4). Overall, 52% of native species were endemic, with 23%, 39% and 68% for freshwater, marine and terrestrial environments respectively (Table 1). The average percent endemicities across taxa were 44%, 36% and 65% per environment respectively (Figure 4).

### Dispersal traits

Microscopic and marine taxa tended to have lower levels of endemicity (Table 2). In addition, benthic marine taxa have greater endemicity than pelagic. A lower proportion of terrestrial taxa occur at lower levels of endemicity (Table 2).

**Figure 5.**
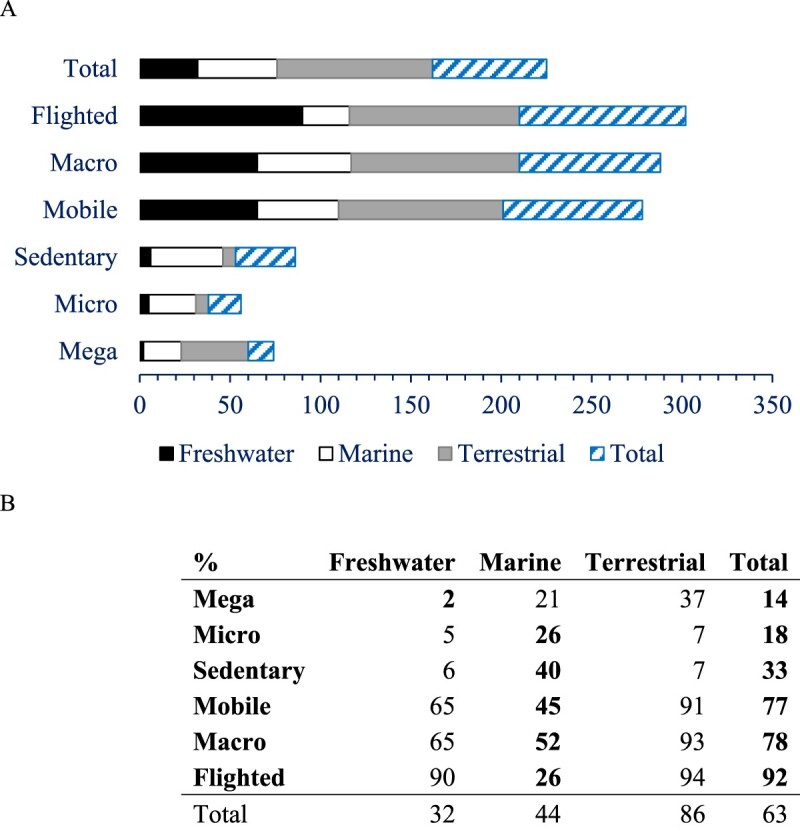
The average percent endemicity of taxa grouped by traits across (**A**) freshwater (black bar), marine (hollow bar), terrestrial (grey bar) and all (hatched bar) taxa recorded in New Zealand as a bar chart and (**B**) table. Because the totals are based on the number of species they are not the same as the average of percentages for each environment.

Microscopic taxa and megafauna had the lowest endemicity, followed by sedentary taxa, especially for freshwater taxa (Figure 5). Mobile, flighted and macro taxa had the highest endemicity across all environments, with 91-94% of these terrestrial trait groups endemic (Table 2).

**Figure 6.**
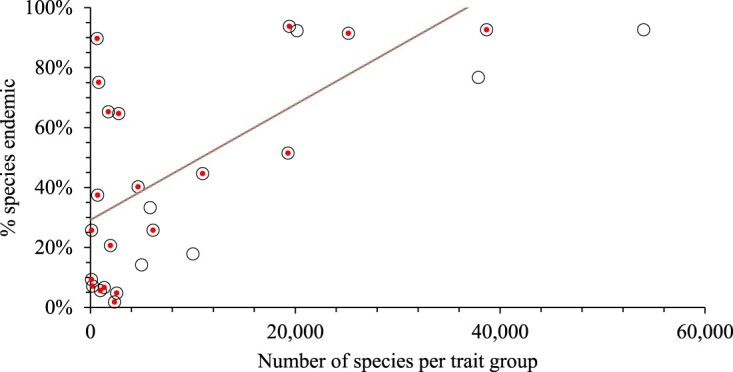
The relationship between percent endemicity to the number of species in each of the trait groups by environment in Figure 5B table of body size, pelagic-benthic, mobile-sedentary and flight (*n* = 18, red dots, R^2^ 0.37). Hollow circles are the totals across the environments and excluded from the correlation to avoid double counting.

There was exceptionally high endemicity of flighted freshwater and terrestrial taxa, suggesting poor dispersal by flight (Table 2, Figure 5). While endemicity was generally lower in the marine environment, it was four to five times higher for microscopic and sedentary animal taxa than in freshwater and terrestrial environments (Figure 5). Also, in the marine environment, the 97 species of entirely pelagic and 803 species of entirely benthic taxa had 9% and 75% endemicity respectively. While there was no significant correlation between percent endemicity of taxa and their regional and global species, there was when taxa were grouped by these traits (Figure 6).

## Discussion

### Environment

The results confirmed the expected lower endemicity of marine compared to terrestrial species. This reflects the natural isolation of the land by the ocean while an artificial boundary, the EEZ, is used to bound the marine biota. Furthermore, globally, marine species endemicity decreases with distance from the coast and depth as the environment becomes more stable and homogenous (Costello et al. 2017, 2018; Sayre et al. 2017). Thus, marine species generally inhabit wider depth and geographic ranges with depth, and marine species endemicity is greater in shallow depths.

Freshwater endemicity (excluding freshwater insects) was similar or lower to marine which contradicted expectations. Because most freshwater habitats are temporary on evolutionary timescales, their fauna and flora need to have effective dispersal, whether aerial, through coastal seas, or by attachment to animals (phoresy). For example, Dehling et al. (2010) found species associated with apparently more isolated standing waters (e.g. lakes, ponds) were more widely distributed than species in running waters. Thus, the lower endemicity of freshwater than terrestrial species in New Zealand seems due to the temporary nature of their habitats, and consequently their biota have good dispersal mechanisms.

### Body size

The higher endemicity and species richness of benthic compared to pelagic, and macrofauna compared to microscopic and mega-fauna, were also confirmed. Thus, the largest and smallest species tend to be more cosmopolitan and thus likely to have higher gene flow and fewer species, explaining their previously reported species richness globally (Costello et al. 2013b). In the ocean, the smallest and largest pelagic taxa are the most widespread and have fewer species compared to macrofauna (Costello et al. 2017). However, 37% endemicity for terrestrial megafauna in New Zealand is not low and reflects long term isolation and distance from other continents.

### Macrofauna

That macrofauna dominate species richness from local to global scales is clear (Costello et al. 2013b). The New Zealand data show this size group also has exceptionally high endemicity, 78% overall and 65%, 52% and 93% across freshwater marine and terrestrial environments respectively (Figure 5). Yet, the fact that most arthropods are mobile, and generally most insects can fly, could lead to high dispersal and gene flow and low speciation rates. If this is true, then evidently other factors are driving diversification (speciation minus extinctions) apart from potential mobility. The ability of mobile macrofauna to specialise in geographically scattered microhabitats and food sources, including becoming parasitic, opens opportunities for ecological niche differentiation (Costello and Chaudhary 2017). However, they are at high risk of predation by highly mobile vertebrates. The need to avoid predation is apparent in the nocturnal emergence of benthic freshwater (so called ‘freshwater drift’ in rivers), marine (including benthos emergence and vertical migration of pelagic species) and terrestrial arthropods (McLeod and Costello 2017). Thus, in addition to niche specialisation macrofauna are under continuous pressure to reduce predation risk, which is likely a stronger driver of selection than food supply because death limits evolution more than going hungry (the ‘life-dinner’ hypothesis, Dell et al. 2011). Thus, the high endemicity of mobile macrofauna may be a consequence of opportunities for niche specialisation and the need to limit predation.

Preliminary support for a role of predation is that marine macro-epi-benthic diversity is lower in the tropics than polar latitudes, and inversely correlated with fish species’ abundance (Edgar et al. 2017). Furthermore, the diversity of amphipod crustaceans seems surprisingly low in the Coral Triangle, considered the most rich marine region for fish and corals, compared to higher latitudes (Arfianti and Costello 2021). There is growing experimental evidence of higher tropical predation rates in marine pelagic and benthic environments (Rodemann and Brandl 2017; Roesti et al. 2020; Whalen et al. 2020; Freestone et al. 2021; Ashton et al. 2022), and this seems likely to be the case in terrestrial and freshwater environments for the same reasons despite the variable evidence in the literature (Moles and Ollerton 2016). However, predation is only one selection pressure, and the continuously warm tropical climate provides better growing conditions than in the high latitudes where winter dampens growth, and larger populations and shorter generation times provide more opportunities for speciation regardless of predation pressure (reviewed in Costello and Chaudhary 2017).

### Flight

The high endemicity of flighted taxa was not expected, as it may be assumed that species that can fly would be widely dispersed. However, this may only hold across continents and nearby islands for terrestrial and freshwater species. New Zealand appears to be too far from other landmasses for regular colonisation by most flying species. Indeed, New Zealand exemplifies the well-known phenomenon of a loss of flight in island insects and terrestrial birds which further restricts their dispersal (e.g. Darwin 1860; Diamond 1984; Costello 1988; Veale et al. 2018; Foster et al. 2021) and geographic range (McCulloch et al. 2017).

### Richness

The number of species per taxon in New Zealand was highly correlated with the number named globally. Positive correlations have similarly been observed between the number of species of trees in the same taxa between continents (Silva de Miranda et al. 2022), and of new species being named across all taxa and the number of named species (Costello et al. 2013b). However, neither New Zealand nor global richness of taxa correlated with percent endemicity of the New Zealand biota. This contradicted expectations because it was hypothesised that if a taxon had high endemicity then it should have low dispersal and geneflow, leading to high rates of spatial speciation, and high global species richness. In contrast, when taxa were grouped by the traits of body size and mobility there was a significant relationship between endemicity and richness (Figure 6). These contrasting relationships suggest that the dispersal traits of a taxon are more important in leading to new species evolving (speciation) that its phylogeny. Indeed, major marine and terrestrial lineages have gone extinct in the past, significantly reducing their present species richness, and other taxa diversified (Mayhew et al. 2008; Yasuhara et al. 2020). Nevertheless, the variability in the endemicity-richness relationships indicates that either the underlying data are at too coarse a taxonomic resolution, are not sufficiently complete, and/or that other traits and factors are also important in addition to dispersal.

## Conclusion

The relative endemicity of Aotearoa New Zealand’s biota supports the hypothesis that taxa with generally wider dispersal have lower endemicity and fewer species overall, including marine compared to terrestrial, and microscopic compared to macrofauna. Freshwater biota, excluding insects, also have low endemicity reflecting their need to be able to colonise habitats which are ephemeral on evolutionary timescales. While individual megafauna may travel large distances across continental landmasses and if marine, criss-cross the ocean during their lifetime, the remoteness of New Zealand has led to exceptionally high percent endemicity of its terrestrial megafauna, including birds. The mobility and size of macrofauna appears to have enabled great niche specialisation, and predation pressure may simultaneously have limited dispersal. The percent endemicity of these dispersal related traits better predicted species richness than phylogeny as indicated by their taxonomic classifications.

## Supplementary Material

Supplemental material
